# Supplementary material for: Clinical Outcomes Following Re-Operations for Intracranial Meningioma
Source: Cancers (Basel). 2021 Sep 24;13(19):4792. doi: 10.3390/cancers13194792 (PMC8507983; doi:10.3390/cancers13194792)
Supplement: Supplementary file 1 [file cancers-13-04792-s001.zip › cancers-1374324-supplementary.pdf]

Table S1. Reliability analysis for tumour volumes at diagnosis. Intra- and Inter-rater ICC values are included and demonstrate agreeability between independent researchers. 95% confidence intervals for the values are included.

Inter- and Intra-rater Reliability Analysis		
	**Correlation Coefficient**	**95% Confidence Interval**
Inter-Class	0.93	0.86-0.97
Intra-Class	0.97	0.93-0.98

Table S2. Definitions of Landriel-Ibañez classification of post-neurosurgical complications [14].

Landriel-Ibañez Classification Grades	
**Grade 1**	Non-life-threatening deviation from normal post-op course, not requiring invasive treatment
**Grade 1a**	Complication requiring no drug treatment
**Grade 1b**	Complication requiring drug treatment
**Grade 2**	Complication requiring invasive treatment such as surgical, endoscopic, or endovascular interventions
**Grade 2a**	Complication requiring intervention without general anaesthesia (Local anaesthetic)
**Grade 2b**	Complication requiring intervention with general anaesthetic
**Grade 3**	Life-threatening complications requiring management in ICU
**Grade 3a**	Complication involving single organ failure
**Grade 3b**	Complication involving multiple organ failure
**Grade 4**	Complication resulting in death

Table S3. Definitions of Clavien-Dindo classification of post-surgical complications [15].

Clavien-Dindo Classification	
**Grade 1**	Any deviation from the normal postoperative course without the need for pharmacological treatment or surgical, endoscopic, and radiological interventions<br>Allowed therapeutic regimens are: drugs as antiemetics, antipyretics, analgesics, diuretics, electrolytes, and physiotherapy. This grade also includes wound infections opened at the bedside
**Grade 2**	Requiring pharmacological treatment with drugs other than such allowed for grade I complications<br>Blood transfusions and total parenteral nutrition are also included
**Grade 3**	Requiring surgical, endoscopic or radiological intervention
**Grade 3a**	Intervention not under general anaesthesia
**Grade 3b**	Intervention under general anaesthesia
**Grade 4**	Life-threatening complication (including CNS complications) requiring IC/ICU management
**Grade 4a**	Single organ dysfunction (including dialysis)
**Grade 4b**	Multiorgan dysfunction
**Grade 5**	Death of a patient

Table S4. Showing univariate and multivariate analysis steps. Values highlighted in green are ≤0.2 for univariate, or ≤0.05 for multivariate analysis. ‡ Continuous variable. † Binary variable, reference category for these variables is always no. • Where multiple categories exist for a variable the lowest P value within the set has been included.

Variable Name (Reference Category)	Univariate P Value (≤0.2)	Multivariate P Value (≤0.05)
Age at Re-Operation‡	0.606	
Sex (Female)	0.902	
Radiation Induced Meningioma†	0.999	
ACCI at Diagnosis (ACCI 0)	0.691•	
ICOM Classification (Convexity)	0.325•	
Skull Base†	0.629	
Oedema†	0.341	
Hyperintensity†	0.703	
Tumour Volume at Presentation‡	0.971	
Simpson Grade at Initial Operation (Grade 1)	0.337•	
Residual Tumour Volume at Initial Operation‡	0.873	
WHO Grade at Initial Operation (Grade 1)	0.228•	
Worsened Performance Status Following Initial Operation†	0.999	
Adjuvant Radiation After Initial Operation†	0.999	
Post-Operative Complication after Initial Operation†	0.445	
Pre-Operative Performance Status at Re-Operation (Performance Status 0)	0.999•	
Simpson Grade at Re-Operation (Grade 1)	0.487•	
Adjuvant Radiation After Re-Operation†	0.309	
WHO Grade at Re-Operation (Grade 1)	0.847•	
Number of Complications After Re-Operation‡	0.013	0.029
Time to First Recurrence‡	0.022	0.044
Volume at First Recurrence‡	0.475	
Incidental at First Recurrence†	0.343	
Headache at First Recurrence†	0.200	0.437
Seizure at First Recurrence†	1.0	
Limb Weakness at First Recurrence†	1.0	
Limb Sensory Change at First Recurrence†	0.554	
Cranial Nerve Deficit at First Recurrence†	0.707	
Cognitive Deficit at First Recurrence†	1.0	
Psychiatric Manifestation at First Recurrence †	1.0	
